# Association of Plasma Tau With Mortality and Long-term Neurocognitive Impairment in Survivors of Pediatric Cerebral Malaria and Severe Malarial Anemia

**DOI:** 10.1001/jamanetworkopen.2021.38515

**Published:** 2021-12-10

**Authors:** Dibyadyuti Datta, Paul Bangirana, Robert O. Opoka, Andrea L. Conroy, Katrina Co, Caitlin Bond, Yi Zhao, Keisuke Kawata, Andrew J. Saykin, Chandy C. John

**Affiliations:** 1 Ryan White Center for Pediatric Infectious Disease and Global Health, Indiana University School of Medicine, Indianapolis; 2 Department of Psychiatry, Makerere University College of Health Sciences, Kampala, Uganda; 3 Department of Paediatrics and Child Health, Makerere University College of Health Sciences, Kampala, Uganda; 4 Department of Biostatistics and Health Sciences, Indiana University School of Medicine, Indianapolis; 5 Department of Kinesiology, Indiana University School of Public Health–Bloomington, Bloomington; 6 Program in Neuroscience, The College of Arts and Sciences, Indiana University, Bloomington; 7 Indiana Alzheimer’s Disease Research Center and Department of Radiology and Imaging Sciences, Indiana University School of Medicine, Indianapolis; 8 Division of Global Pediatrics, University of Minnesota Medical School, Minneapolis

## Abstract

**Importance:**

Cerebral malaria (CM) and severe malarial anemia (SMA) are associated with persistent neurocognitive impairment (NCI) among children in Africa. Identifying blood biomarkers of acute brain injury that are associated with future NCI could allow early interventions to prevent or reduce NCI in survivors of severe malaria.

**Objective:**

To investigate whether acutely elevated tau levels are associated with future NCI in children after CM or SMA.

**Design, Setting, and Participants:**

This prospective cohort study was conducted at Mulago National Referral Hospital in Kampala, Uganda, from March 2008 to October 2015. Children aged 1.5 to 12 years with CM (n = 182) or SMA (n = 162) as well as community children (CC; n = 123) were enrolled in the study. Data analysis was conducted from January 2020 to May 2021.

**Exposure:**

CM or SMA.

**Main Outcomes and Measures:**

Enrollment plasma tau levels were measured using single-molecule array detection technology. Overall cognition (primary) and attention and memory (secondary) *z* scores were measured at 1 week and 6, 12, and 24 months after discharge using tools validated in Ugandan children younger than 5 years or 5 years and older.

**Results:**

A total of 467 children were enrolled. In the CM group, 75 (41%) were girls, and the mean (SD) age was 4.02 (1.92) years. In the SMA group, 59 (36%) were girls, and the mean (SD) age was 3.45 (1.60) years. In the CC group, 65 (53%) were girls, and the mean (SD) age was 3.94 (1.92) years. Elevated plasma tau levels (>95th percentile in CC group; >6.43 pg/mL) were observed in 100 children (55%) with CM and 69 children (43%) with SMA (*P* < .001). In children with CM who were younger than 5 years, elevated plasma tau levels were associated with increased mortality (odds ratio [OR], 3.06; 95% CI, 1.01-9.26; *P* = .048). In children with CM who were younger than 5 years at both CM episode and follow-up neurocognitive testing, plasma tau levels (log_10_ transformed) were associated with worse overall cognition scores over 24-month follow-up (β = −0.80; 95% CI, −1.32 to −0.27; *P* = .003). In children with CM who were younger than 5 years at CM episode and 5 years or older at follow-up neurocognitive testing, plasma tau was associated with worse scores in attention (β = −1.08; 95% CI, −1.79 to −0.38; *P* = .003) and working memory (β = −1.39; 95% CI, −2.18 to −0.60; *P* = .001).

**Conclusions and Relevance:**

In this study, plasma tau, a marker of injury to neuronal axons, was elevated in children with CM or SMA and was associated with mortality and persistent NCI in children with CM younger than 5 years.

## Key Points

**Question:**

Are plasma tau levels associated with long-term neurocognitive impairment in pediatric cerebral malaria or severe malarial anemia?

**Findings:**

In this cohort study of 467 children in Uganda, including children with cerebral malaria and severe malarial anemia as well as asymptomatic community children, plasma tau levels were higher in children with cerebral malaria or severe malarial anemia compared with asymptomatic community children. Plasma tau levels were associated with increased mortality and worse neurocognitive outcomes in children with cerebral malaria at younger than 5 years.

**Meaning:**

These findings suggest that plasma tau may serve as a brain injury biomarker in 2 clinically diverse forms of pediatric severe malaria and may identify children at risk of developing long-term neurocognitive impairment following cerebral malaria.

## Teaser

This cohort study investigates whether acutely elevated tau levels are associated with future neurocognitive impairment in children after cerebral malaria or severe malarial anemia.

## Introduction

Injury to neuronal axons can occur in acute disorders, such as traumatic brain injury (TBI),^1^ and in infections, such as cerebral malaria (CM),^2^ where it is associated with persistent neurocognitive impairment (NCI).^3,4^ Injury to a child’s brain when complex neural networks are being formed and structured can have lasting consequences, yet there are limited studies of axonal injury in children. Identifying biomarkers that can gauge the initial severity of injury and predict NCI in infection-mediated brain injury, as in CM, is critical to interrupting future NCI among the millions of affected children in malaria-endemic countries.^5^

CM occurs in *Plasmodium falciparum* infection with coma when infected erythrocytes sequester in the brain microvasculature, leading to ischemic and/or hypoxic injury,^6,7^ hemolysis and cellular damage,^7,8^ inflammation,^9,10^ coagulation,^11^ and endothelial and/or blood-brain barrier dysfunction.^8^ CM is associated with persistent NCI in survivors.^12^ Severe malarial anemia (SMA), defined as hemoglobin levels of 5 g/dL or less (to convert to grams per liter, multiply by 10) with *P falciparum* infection, is the most common form of severe malaria in children in Africa.^13^ Although children with SMA do not have acute neurologic deficits, they do develop long-term NCI.^14^ Several infection-induced pathophysiologic features of CM and SMA overlap with other neurologic disorders. For example, in TBI, coagulopathy, elevated cellular injury marker lactate dehydrogenase,^15,16^ and increased expression of endothelial/blood-brain barrier dysfunction markers^17,18^ complicate focal axonal injury, which can impair cognitive functions.^19^

Tau is a marker of injury to neuronal axons that is predominantly expressed by neurons and localized within axons where it stabilizes microtubule networks.^20^ In CM, as in various neurologic disorders including TBI and Alzheimer disease, elevated cerebrospinal fluid (CSF) levels of tau have been associated with persistent NCI.^4,21,22^ The use of tau as a biomarker of neurologic injury was previously restricted to populations where CSF is accessible, due to its low abundance in blood and lack of sensitive detection tools. As a consequence, noninvasive assessment of axonal injury has not been possible in disorders like SMA, where CSF collection is not clinically indicated. With the development of highly sensitive detection assays for blood-based tau,^23^ plasma tau levels have been shown to reflect the severity of diffuse axonal injury^24,25^ and persistent NCI in neurologic disorders.^26^ A recent study published reliable reference ranges for plasma tau levels in pediatric TBI in a high-income country setting.^27^ The promising utility of plasma tau as a marker of axonal injury and the global health implications of such injury in pediatric populations warrants the question of whether plasma tau can serve as a proxy marker of NCI in severe infections like malaria that continue to adversely affect the developmental potential of millions of children in malaria-endemic low- and middle-income countries.

In the current study, we used a highly sensitive single-molecule assay to measure plasma tau levels in a prospective cohort study of children in Uganda who were hospitalized with CM or SMA as well as community children and evaluated the association of plasma tau with cognitive functions during 24 months of follow-up. We also evaluated potential mechanisms that could lead to axonal injury and the association of plasma tau with mortality. We hypothesized that plasma tau would serve as a proxy biomarker for identifying children at risk of NCI after CM and SMA.

## Methods

The study was approved by the institutional review boards at Makerere University School of Medicine and the University of Minnesota and by the Uganda National Council for Science and Technology. Written informed consent was obtained from parents or guardians of study participants. The study was conducted following the Strengthening the Reporting of Observational Studies in Epidemiology (STROBE) reporting guidelines for observational studies.

### Study Design, Participants, and Assessments

This study was performed at Mulago Hospital in Kampala, Uganda, from March 2008 to October 2015. Children were eligible if they were aged between 1.5 and 12 years. CM was defined as follows: coma (Blantyre Coma Score ≤2 or Glasgow Coma Score ≤8), *P falciparum* on blood smear, and no other known cause of coma. SMA was defined as follows: *P falciparum* on blood smear and hemoglobin level of 5 g/dL or lower. Children were managed according to the Ugandan Ministry of Health treatment guidelines at the time of the study. A reference group of age-matched community children (CC) with no active illness were recruited from the extended family or household compound of children with CM or SMA. Children underwent a medical history and physical examination at enrollment. Anthropometric *z* scores were generated using age-appropriate World Health Organization (WHO) reference standards (eMethods in the Supplement). Age-appropriate versions of the WHO Home Observation for the Measurement of the Environment were administered and converted to *z* scores.^28^ Socioeconomic status was measured using a previously published validated scoring system.^29^ Additional assessments are described in eMethods in the Supplement.

### Plasma Tau Measurements, Clinical Laboratory Tests, and Immunoassays

Sample collection, processing, and storage details are described in eMethods in Supplement. Plasma total tau levels were measured between August and September 2018 using a highly sensitive single-molecule array (Simoa) HD-1 analyzer (Quanterix), with a lower limit of tau detection at 0.024 pg/mL.^30^ The assay uses monoclonal antibodies that bind both normal and phosphorylated tau and detect all tau isoforms. Samples were tested blinded to participant details and 10% of samples were run in duplicate (eMethods in the Supplement). Current pediatric reference ranges for plasma tau are available from a single study of Canadian children.^27^ We established a pediatric reference range where tau levels greater than the 95th percentile of children in the CC group were considered elevated. CSF tau testing details have been previously described.^4^ To elucidate possible mechanisms leading to increased plasma tau in severe malaria, parasite factors, clinical risk factors, and clinical laboratory tests associated with disease severity in CM or SMA (eMethods in the Supplement) were compared in children with vs without elevated tau. Furthermore, we evaluated associations between plasma markers of endothelial/brain-barrier dysfunction (eMethods in the Supplement) and plasma tau levels.

### Neurologic and Cognitive Assessments

Neurologic and cognitive assessments in children with CM or SMA were performed at discharge (neurologic testing) or 1 week after discharge (cognitive testing) and at the 6-, 12-, and 24-month follow-up visits. Children in the CC group underwent cognitive testing at enrollment and follow-up. A neurologic deficit was defined as presence of motor or cranial nerve deficits; ataxia; movement, speech, or visual disorders; or behavioral problems. Different cognitive testing batteries were used for children younger than 5 years and those 5 years older, so results are presented for (1) children younger than 5 years at malaria episode and follow-up testing, (2) children younger than 5 years at malaria episode who crossed age strata and were 5 years or older at follow-up testing, and (3) children 5 years or older at the time malaria episode and follow-up testing. For children younger than 5 years, the Mullen Scales of Early Learning was used to measure cognition.^31^ Scores from fine motor, visual reception, receptive language, and expressive language scales were summed to give the early learning composite score, a measure of overall cognition. Attention was assessed using the Early Childhood Vigilance Test.^32^ Associative memory was assessed using the Color Object Association Test.^33^ In children 5 years or older, the Kaufman Assessment Battery for Children, second edition (KABC-2), was used to measure overall cognition, with summary mental processing index as the primary outcome.^34^ Attention was assessed using the Test of Variables of Attention, with D prime measure as the primary outcome.^35^ The KABC-2 subtest for sequential processing was used to assess working memory. These age-range specific cognitive assessments have been validated and used in prior studies of children in Uganda.^12,14,36^ Age-adjusted *z* scores were created using scores of the children in the CC group, as previously described.^37^

### Statistical Analysis

Analyses were done using Stata version 14.0 (StataCorp). Differences in continuous measures were evaluated using Wilcoxon rank-sum test (2 groups) or Kruskal-Wallis test (>2 groups). Differences in categorical variables were measured using the Pearson χ^2^ test. The a priori level of significance was .05 with 2-sided hypothesis tests. Spearman rank analysis was used to evaluate correlation between CSF and plasma tau in the CM group. Logistic or linear regression, adjusting for age and sex, were used to evaluate associations between plasma tau concentrations and dichotomous or continuous outcomes, respectively. Tau concentrations and other continuous variables not normally distributed were log_10_ transformed for inclusion in regression models. To evaluate the discriminatory ability of plasma tau to estimate neurologic deficits (ND) over time, nonparametric receiver operating characteristic curve analysis was used, and the area under the curve (AUC) was compared. Linear mixed effects (LME) models^4^ were used to assess the association between plasma tau and cognitive *z* scores at each point when observations within participants were correlated using a participant-specific intercept, and time points were treated as categorical variables. LME models were adjusted for sociodemographic factors that were significantly different between the disease groups and control group (Table). Cognitive outcome analysis was not adjusted for multiple comparisons given that only a single primary outcome and 2 secondary outcomes were evaluated.

**Table.** Baseline Sociodemographic Characteristics of Children in the Study With Admission Plasma Tau Levels Measured

Characteristic	No. (%)			*P* value^b^
	Children with cerebral malaria (n = 182)^a^	Children with severe malarial anemia (n = 162)^a^	Community control children (n = 123)^a^	
Age, median (IQR), y	3.54 (2.59 to 4.95)	2.89 (2.10 to 4.50)	3.58 (2.60 to 4.59)	.005^c^
Age group, median (IQR), y				
<5	3.09 (2.33 to 3.90)	2.84 (2.04 to 3.53)	3.22 (2.49 to 3.92)	.20
≥5	6.45 (5.99 to 7.31)	5.95 (5.29 to 6.99)	7.01 (5.95 to 8.65)	
Sex				
Girls	75 (41.21)	59 (36.42)	65 (52.85)	.02^d^
Boys	107 (58.79)	103 (63.58)	58 (47.15)	
For-age *z* score, median (IQR)				
Height	−1.20 (−1.89 to −0.18)	−1.35 (−2.3 to −0.58)	−1.36 (−1.87 to −0.55)	0.11
Weight	−1.0 (−1.74 to −0.38)	−1.28 (−2.12 to −0.48)	−0.83 (−1.37 to −0.18)	.001^d^
Socioeconomic status score, median (IQR)^e^	9 (8 to 12)	9 (7 to 11)	9 (8 to 12)	.28
Home environment *z *score, median (IQR)	0.04 (−0.86 to 0.74)	−0.06 (−0.72 to 0.67)	0.21 (−0.72 to 0.85)	.47
Maternal education				
Primary 6 or lower	64 (35.16)	61 (37.65)	34 (27.64)	.33
Primary 7	33 (18.13)	35 (21.60)	32 (26.02)	
Secondary	58 (31.87)	59 (33.42)	50 (40.65)	
Unknown	27 (14.84)	7 (4.32)	7 (5.69)	
Paternal education				
Primary 6 or lower	33 (18.13)	36 (22.22)	21 (17.07)	.75
Primary 7	27 (14.86)	25 (15.43)	24 (19.51)	
Secondary	72 (39.56)	77 (47.53)	63 (51.22)	
Unknown	50 (27.47)	24 (14.81)	15 (12.20)	
Child preschool education, No./total No. (%)	63/162 (38.89)	47/157 (29.94)	48/122 (39.34)	.16

^a^ Data available and presented for surviving children who had a home visit.

^b^ Kruskal-Wallis test for continuous variables; Pearson χ^2^ test for categorical variables.

^c^ Children with severe malarial anemia differ from those with cerebral malaria and those in community control group. Pairwise comparisons conducted using Wilcoxon rank-sum test.

^d^ Children with cerebral malaria and severe malarial anemia differ from children in community control group. Pairwise comparisons conducted using Wilcoxon rank-sum test for continuous and χ^2^ test for categorical variables.

^e^ The socioeconomic composite score ranges from 0 to 12, with lower values indicating worse socioeconomic status.

## Results

### Baseline Sociodemographic Characteristics

**Figure 1.**
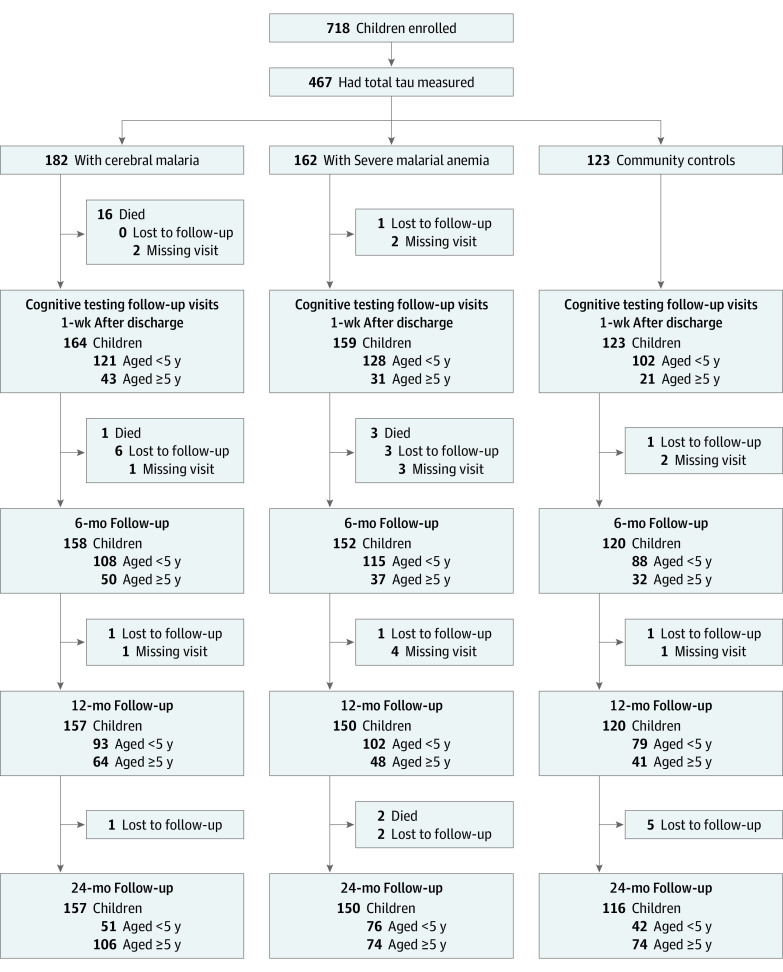
Study Participant Flow Diagram

Of the 718 children in the parent study, 467 with sufficient plasma available for tau testing were included (Figure 1 and Table). Children included were representative of the full cohort (published in Bangirana et al^14^). Overall, there were 182 children with CM (75 [41%] girls; mean [SD] age, 4.02 [1.92] years); 162 with SMA (59 [36%] girls; mean [SD] age, 3.45 [1.60] years), and 123 in the CC group (65 [53%] girls; mean [SD] age, 3.94 [1.92] years). Weight-for-age adjusted *z* scores were significantly lower in children with CM or SMA compared to CC. Other factors were similar across study groups (Table).

### Plasma Tau Levels in CM and SMA Groups vs CC Groups and Correlation of Plasma and CSF Tau in CM Groups

**Figure 2.**
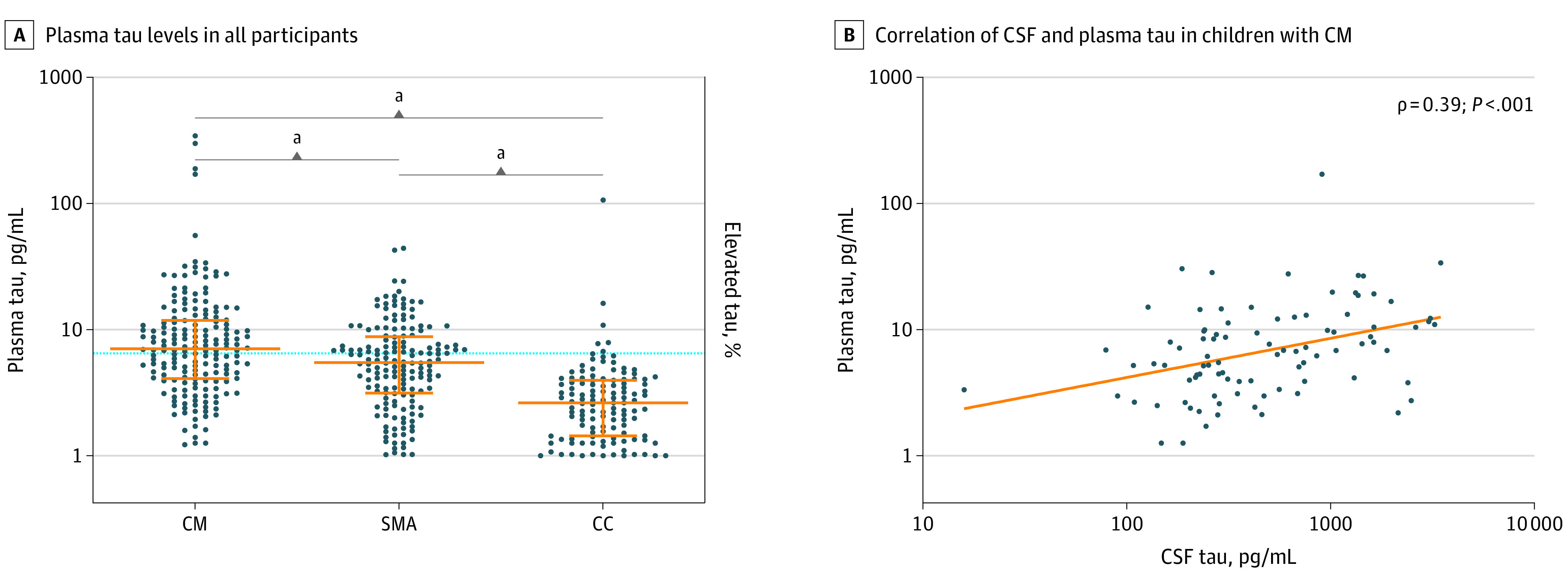
Plasma Tau Levels in All Study Participants and Cerebrospinal Fluid (CSF) and Plasma Tau Correlations in Children with Cerebral Malaria (CM) A, Dots represent tau levels in individual children, and orange lines show median (center line) and IQR (whiskers). The 95th percentile for tau levels in the community children (CC) group (6.43 pg/mL) is indicated by the horizontal line. SMA indicates severe malarial anemia. ^a^*P* < .001.

Tau levels greater than 6.43 pg/mL (the 95th percentile for CC) were considered elevated. Elevated plasma tau levels were observed in 100 children (55%) with CM and 69 children (43%) with SMA compared with 7 children (4.9%) in the CC group (*P* < .001) (Figure 2A). Tau levels were highest in children with CM younger than 5 years compared with children with CM aged 5 years or older or children with SMA (eTable 1 in Supplement). Tau levels did not vary by age in the CC group, validating the use of 95th percentile CC to report elevated tau across age groups. Among the 91 children with CM who had both plasma and CSF tau measured, the levels were correlated (ρ = 0.39; *P* < .001) (Figure 2B).

### Association With Mortality

In the 344 children with CM or SMA, a 1-unit increase in log_10_ plasma tau levels was associated with 5.58-fold increased odds of in-hospital death (95% CI, 1.92-16.23; *P* = .002) and mortality was higher among those with elevated plasma tau levels (12 [7%]) than those without (4 [2.3%]; *P* = .03). As no child aged 5 years or older age died, we conducted analyses in children with CM who were younger than 5 years. Elevated tau was associated with 3.06-fold increased odds of mortality in-hospital (95% CI, 1.01-9.26, *P* = .048).

### Association With Neurologic and Cognitive Outcomes

**Figure 3.**
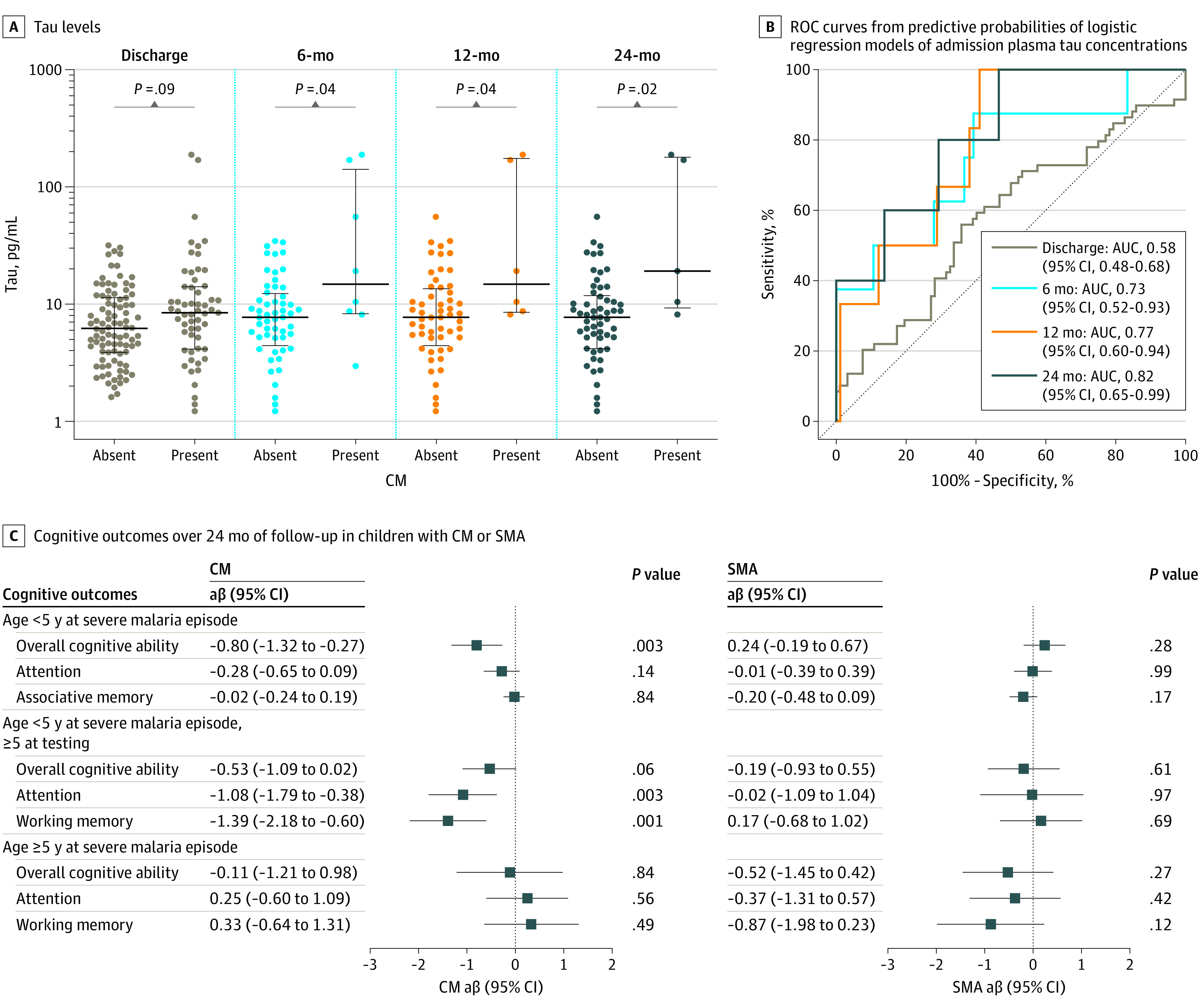
Admission Plasma Tau Associations With Neurologic Deficits in Cerebral Malaria (CM) and Cognitive Impairments in CM or Severe Malarial Anemia (SMA) Over 24 Months of Follow-up A, Levels and nonparametric receiver operating characteristic curve from estimated probabilities of logistic regression models of admission plasma tau concentrations in children with CM without neurologic deficits (absent) and with neurologic deficits (present) at discharge and 6, 12, and 24 months after discharge. Dots represent plasma tau levels in individual children, and black lines show median (center line) and IQR (whiskers). C, Association of admission plasma tau levels (log_10_ transformed) with different cognitive outcome domains over 24 months of follow-up in children younger than 5 years and 5 years or older with CM or SMA. Figure represents the adjusted β (aβ) coefficient and 95% CIs for plasma tau and longitudinal changes in cognitive *z* scores measured at 1 week and 6, 12, and 24 months after discharge. *P* values were obtained using linear mixed effects modeling, in which observations within participants with cognition data available at each time point (Figure 1) were correlated using a participant-specific intercept, and time points were treated as categorical variables. All models were adjusted for sociodemographic factors significantly different between disease groups and the control group (age, sex, and weight-for-height *z* scores). AUC indicates area under the receiver operating characteristic curve.

Elevated plasma tau was associated with a longer duration of coma (in hours) in children with CM (β = 0.14; 95% CI, 0.002-0.28; *P* = .046). Plasma tau levels were not different in 59 children with ND at discharge compared with 102 children without ND. However, children with ND on follow-up had significantly higher admission tau levels compared with those without, and the predictive value of admission plasma tau for ND increased over time, with AUCs of 0.58 (95% CI, 0.48-0.68), 0.73 (95% CI, 0.52-0.93), 0.77 (95% CI, 0.60-0.94), and 0.82 (95% CI, 0.65-0.99) at discharge and 6-, 12-, and 24-month follow-ups, respectively (Figure 3A), despite the small numbers of children with ND in follow-up (8 at 6 months; 6 at 12 months; 5 at 24 months). Children with SMA did not demonstrate ND acutely or in follow-up.

In the 121 children with CM who were younger than 5 years at time of malaria episode, admission plasma tau was associated with worse overall cognition over follow-up (β = −0.80; 95% CI, −1.32 to −0.27; *P* = .003). Among the 65 children younger than 5 years at the time of malaria episode who subsequently crossed the 5-year age mark during follow-up and were tested with the cognitive testing battery for children 5 years or older, increased plasma tau was associated with worse attention (β = −1.08; 95% CI, −1.79 to −0.38; *P* = .003) and working memory (β = −1.39; 95% CI, −2.18 to −0.60; *P* = .001) (Figure 3B). In 42 children with CM who were 5 years or older at the time of malaria episode, and in all children with SMA, there was no association between plasma tau and any cognitive outcome (Figure 3B).

### Association With Clinical Risk Factors in Severe Malaria

**Figure 4.**
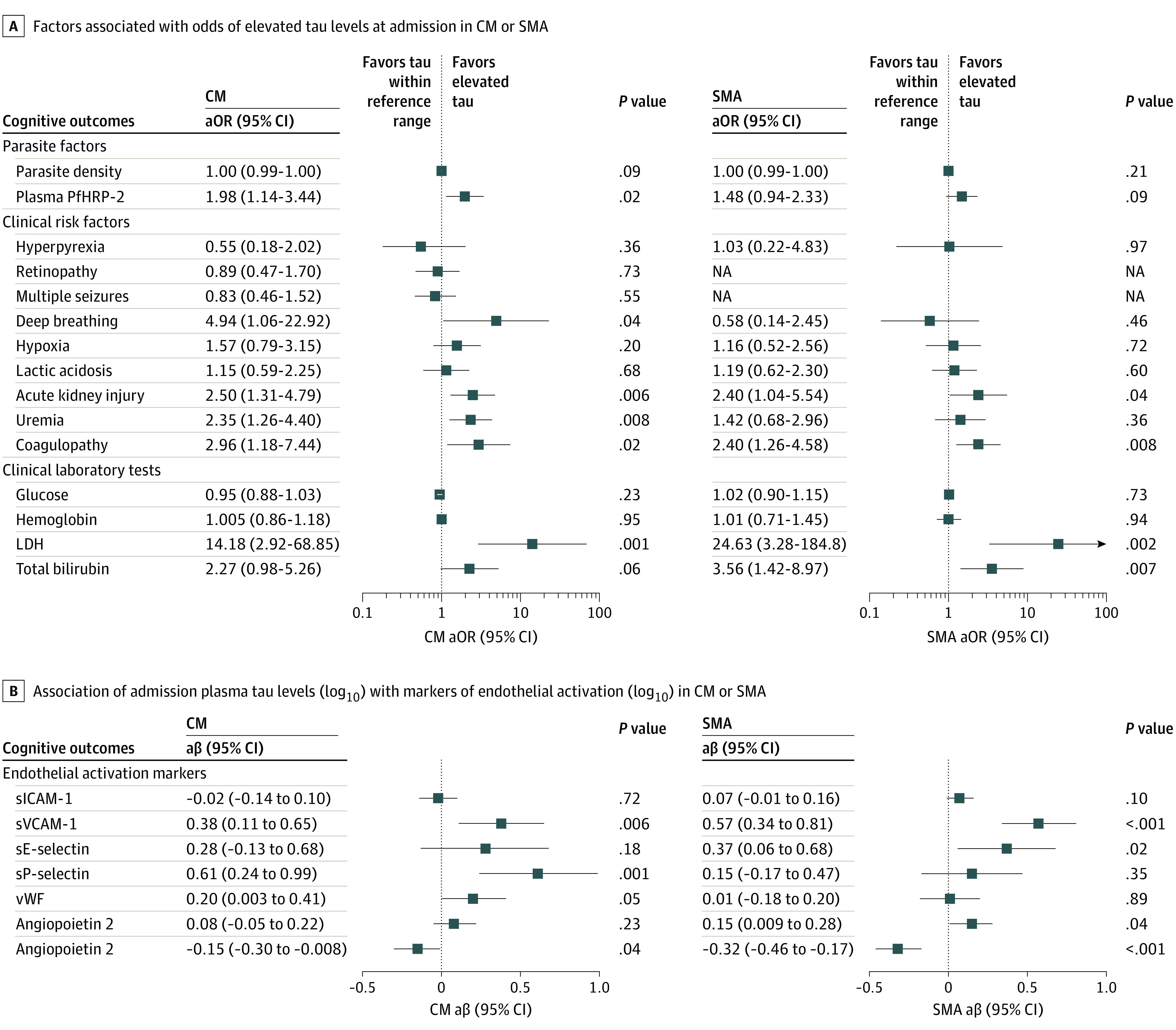
Admission Plasma Tau Associations With Clinical Risk Factors and Endothelial Activation Markers A, Median adjusted odds ratios (aORs) and 95% CIs are shown. *P* values indicate univariable logistic regression analysis. eTable 2 in the Supplement shows differences between factors based on tau levels in the reference range vs elevated. B, Median adjusted β (aβ) coefficient and 95% CI are shown. *P* values indicate univariable linear regression analysis. CM indicates cerebral malaria; LDH, lactate dehydrogenase; PfHRP-2, *Plasmodium falciparum* histidine-rich protein-2; sE-selectin, soluble E-selectin; sICAM-1, soluble intercellular adhesion molecule-1; SMA, severe malarial anemia; sP-selectin, soluble P-selectin; sVCAM-1, soluble vascular cell adhesion molecule-1; vWF, von Willebrand factor.

In either CM or SMA or both, elevated tau was associated with *P falciparum* histidine rich protein (PfHRP-2), a measure of parasite biomass; lactate dehydrogenase (LDH), a marker of cellular damage and hemolysis; total bilirubin, a marker of hemolysis and liver dysfunction; and presence of acute kidney injury, uremia, and coagulopathy (Figure 4A; eTable 2 in the Supplement). In multivariable regression incorporating all factors significant in single-factor analysis, coagulopathy (CM group: adjusted OR [aOR], 3.50; 95% CI, 1.05-11.69; SMA: aOR, 2.28; 95% CI, 1.01-5.14) and elevated LDH levels (CM: aOR, 7.39; 95% CI, 1.11-48.94; SMA: aOR, 18.58; 95% CI, 1.69-203.77) were independently associated with elevated tau (eFigure in the Supplement). Furthermore, elevated plasma levels of endothelial/barrier dysfunction markers soluble vascular cell adhesion molecule 1, E-selectin, and P-selectin as well as decreased levels of angiopoietin 1 were independently associated with plasma tau levels (Figure 4B).

## Discussion

Blood-based biomarkers of neurologic injury, particularly biomarkers that correlate with persistent neurologic or cognitive impairment, could provide valuable information on the extent to which diseases—even those without obvious neurologic findings—cause brain injury. In this study, we found that plasma tau, a marker of injury to neuronal axons, was elevated in children with CM, who present in coma, as well as children with SMA, who have no clinical signs of neurologic disease. Elevated plasma tau levels were associated with persistent NCI in CM but not SMA. Elevated tau was also associated with mortality, which, in this study, was only seen among children with CM. In both disease processes, children with elevated plasma tau had elevated levels of LDH, a marker of cellular injury, and platelet deficiency, resulting from hemolysis, as well as reduced levels of plasma angiopoietin-1, a marker of endothelial stability. Together, the data suggest that axonal injury occurs in both CM and SMA; is more pronounced in CM, in which it is associated with NCI; and is potentially mediated by hemolysis, endothelial activation, and cellular injury.

CSF biomarkers have provided insights into the mechanisms of brain injury in the context of infection or trauma, but lumbar punctures, which are required to obtain CSF, are invasive procedures indicated only when required to rule out specific diagnoses, such as bacterial meningitis. For this reason, blood biomarkers of neurologic injury are urgently needed. The low abundance of neuronal proteins in blood, a lack of assay sensitivity and specificity, and variability in data from enzyme-linked immunosorbent assay–based detection tools^38^ have led to skepticism about the likelihood of establishing sensitive and specific blood biomarkers of brain injury.^39^ Here, we addressed the limitations of using conventional assays to measure plasma tau in severe malaria^40^ by using a single-molecule detection array with sensitivity in the femtomolar range.^23^ Using this novel assay in a large cohort study of children with severe malaria, we found that plasma tau levels were elevated in children with CM and SMA.

The plasma tau levels observed in the children in the CC group were consistent with reference ranges established in a high-income setting.^27^ In children with CM, plasma tau levels were comparable with levels reported in children with moderate TBI,^27^ and in children younger than 5 years, in whom there was the strongest association between plasma tau and NCI, plasma tau levels were comparable with levels reported in severe TBI.^27^ Furthermore, the associations of elevated plasma tau with mortality, longer duration of coma, and persistent neurologic deficits up to 24 months after discharge in the CM group corroborate our previous findings with CSF tau.^4^ These findings support the role of plasma tau as a proxy marker of acute and long-term neurologic complications in children with CM.

Plasma tau was elevated in children with SMA compared with the control group, but levels were lower than those seen in children with CM and consistent with levels seen in children with mild TBI.^27^ In mild TBI, cognitive problems typically do not persist beyond the acute phase of injury,^41^ but children with SMA have persistent NCI at a level similar to children with CM.^14^ However, the lack of association between plasma tau and NCI in children with SMA in the present study suggests that, although axonal injury occurs in SMA, it is less severe than in CM and may be driven by mechanisms other than tau-mediated axonal damage.

The utility of tau as a biomarker of brain injury in severe malaria is strengthened by its association with established pathways of malaria pathogenesis including coagulopathy, cellular injury, and endothelial/blood-brain barrier dysfunction.^8,11^ The associations of plasma tau with infection-driven pathogenic pathways in our study appeared to mimic the cascade of events seen after mechanical injury to the brain in TBI, in which coagulopathy, elevated LDH levels, and endothelial/blood-brain barrier dysfunction^15,16,42^ may permit markers of brain injury like tau to cross into blood circulation.^43^ Our study provides an impetus for evaluation of plasma tau with single-molecule assay methods in other infection-mediated neurologic disorders, to determine the extent of axonal injury and whether plasma tau levels in these conditions are also associated with NCI.

### Limitations

This study has limitations. Our conclusions are drawn from a single study cohort: additional studies conducted globally are needed to confirm these data. The lack of association of plasma tau with NCI in children with SMA suggests the need for further investigations into mechanisms associated with NCI in this population. Finally, the lack of serial tau testing at follow-up points limits our ability to comment on the persistence of axonal injury in this cohort.

## Conclusions

Using a highly sensitive single-molecule assay, we found that children hospitalized with CM or SMA had elevated levels of plasma tau, a marker of injury to neuronal axons, and that tau levels were associated with disease severity, mortality, and persistent NCI in children with CM. Plasma tau shows promise as a reliable, minimally invasive biomarker of neurologic injury that could be used in resource-limited countries to identify children susceptible to persistent NCI. Additional tau testing in healthy children in multiple African countries should be conducted to validate our findings and to establish community-level reference ranges for children in Africa. If validated in additional studies as a biomarker for neurologic injury, a plasma tau point-of-care test could be a valuable tool for identifying children with the highest risk of future NCI and therefore with the greatest needed for acute cognitive rehabilitation.

## Notes

Supplement.
eMethods.
**eTable 1.** Admission CSF and Plasma Tau Concentrations in Children Younger Than 5 Years and 5 Years or Older **eTable 2. **Distribution of Parasite Factors, Clinical Risk Factors, and Levels of Clinical Laboratory Markers by Normal vs Elevated Admission Tau Concentrations in Children With Cerebral Malaria and Severe Malarial Anemia **eFigure. **Multivariate Logistic Regression Analysis of Factors Associated With Normal vs Elevated Admission Tau Concentrations in Children With Cerebral Malaria and Severe Malarial Anemia
eReferences.
