# Supplementary material for: Association of Plasma Tau With Mortality and Long-term Neurocognitive Impairment in Survivors of Pediatric Cerebral Malaria and Severe Malarial Anemia
Source: JAMA Netw Open. 2021 Dec 10;4(12):e2138515. doi: 10.1001/jamanetworkopen.2021.38515 (PMC8665370; doi:10.1001/jamanetworkopen.2021.38515)
Supplement: Supplement. — eMethods. eTable 1. Admission CSF and Plasma Tau Concentrations in Children Younger Than 5 Years and 5 Years or Older eTable 2. Distribution of Parasite Factors, Clinical Risk Factors, and Levels of Clinical Laboratory Markers by Normal vs Elevated Admission Tau Concentrations in Children With Cerebral Malaria and Severe Malarial Anemia eFigure. Multivariate Logistic Regression Analysis of Factors Associated With Normal vs Elevated Admission Tau Concentrations in Children With Cerebral Malaria and Severe Malarial Anemia eReferences. [file jamanetwopen-e2138515-s001.pdf]

Supplemental Online Content

Datta D, Bangirana P, Opoka RO, et al. Association of plasma tau with mortality and long-term neurocognitive impairment in survivors of pediatric cerebral malaria and severe malarial anemia. *JAMA Netw Open.* 2021;4(12):e2138515. doi:10.1001/jamanetworkopen.2021.38515

eMethods.

eTable 1. Admission CSF and Plasma Tau Concentrations in Children Younger Than 5 Years and 5 Years or Older

eTable 2. Distribution of Parasite Factors, Clinical Risk Factors, and Levels of Clinical Laboratory Markers by Normal vs Elevated Admission Tau Concentrations in Children With Cerebral Malaria and Severe Malarial Anemia

eFigure. Multivariate Logistic Regression Analysis of Factors Associated With Normal vs Elevated Admission Tau Concentrations in Children With Cerebral Malaria and Severe Malarial Anemia

eReferences.

This supplemental material has been provided by the authors to give readers additional information about their work.

© 2021 Datta D et al. *JAMA Network Open.*

eMethods

1. Study Design and Participants

This prospective cohort study was performed at Mulago Hospital in Kampala, Uganda, from 2008-2015. Children were eligible if they were between 1.5 and 12 years of age. CM was defined as: 1) coma (Blantyre Coma Score [BCS] ≤2 or Glasgow Coma Score [GCS] ≤8), 2) *P. falciparum* on blood smear, and 3) no other known cause of coma (e.g., meningitis, a prolonged postictal state or hypoglycemia-associated coma reversed by glucose infusion). Duration of coma was defined as time from admission until the child regained full consciousness (BCS=5 or GCS=15). SMA was defined as the presence of *P. falciparum* on blood smear in children with a hemoglobin level ≤5g/dL. A reference group of age-matched community children (CC) with no chronic or active illness were recruited from the extended family, or household compound areas of children with CM or SMA. Exclusion criteria for all children included: 1) known chronic illness requiring medical care; 2) known developmental delay; or 3) history of coma, head trauma, hospitalization for malnutrition, or cerebral palsy. Additional exclusion criteria for children with SMA included: 1) impaired consciousness on physical examination; 2) other clinical evidence of CNS disease; or 3) ≥ 1 seizures prior to admission. Additional exclusion criteria for CC included: 1) illness requiring medical care within the previous 4 weeks or 2) major medical or neurologic abnormalities on screening physical examination. Children were managed according to the Ugandan Ministry of Health treatment guidelines at the time of the study, which included intravenous infusion of quinine hydrochloride over a 4-hr period for the treatment of severe malaria, repeated every 8-hr until the child could take oral medication (quinine or artemether-lumefantrine). Towards the end of the study, the treatment of severe malaria shifted to the use of parenteral artemisinin-based therapies following the 2011 World Health Organization (WHO) recommendation of injectable artesunate as the first-line treatment for severe malaria.

2. Ethics Review and Informed Consent

Written informed consent was obtained from parents/guardians of study participants. Ethical approval was granted by the Institutional Review Boards at Makerere University School of Medicine (SOMREC, reference number: 2008-033, date of first approval: April 7, 2008) and the University of Minnesota (IRB Code Number: 0808M27022, date of first approval: March 31, 2008). The study was also approved by the Uganda National Council for Science and Technology (UNCST, reference number: HS432, date of first approval, May 16, 2008).

3. Demographic and Clinical Assessments

All children underwent a medical history and physical examination at enrollment. Nutritional status was assessed using the 2006 WHO growth standards for children <5 years and 2007 WHO growth references for children ≥5 years and reported as height-for-age z-scores (HAZ) or weight-for-age z-scores (WAZ). Emotional stimulation in the home was measured using age-appropriate versions of the Home Observation for the Measurement of the Environment.[1] Socioeconomic status (SES) was measured using a validated scoring system previously published.[2] Hypoglycemia was treated with a dextrose bolus administered intravenously. HIV testing was performed according to the Uganda National HIV testing algorithm after obtaining consent from the parent or guardian. Sickle cell genotype was determined as described.[3]

4. Sample Collection, Processing, Storage and Clinical Laboratory Testing

After obtaining consent from parents or guardians for participation in the study, a blood draw was performed for study specific procedures. Within 2 hours of sample collection, blood samples were sent to the performing laboratory for processing and storage. If a sample was collected after hours, it was stored at 4-8°C until the following morning when it was processed and EDTA or lithium heparin anti-coagulated plasma samples were stored at -80ºC until testing. Giemsa-stained peripheral blood smears were assessed for *Plasmodium* species and quantified using standard protocols. Children were assessed for malarial retinopathy using indirect ophthalmoscopy as previously described.[4] *P. falciparum* histidine rich protein (PfHRP-2) in plasma was quantified using the Malaria Ag CELISA (Cellabs, Brookvale, Australia), and parasite biomass was calculated as described.[5] Complete blood count (CBC) to enumerate hemoglobin, platelets, and whole blood cells (WBCs) was performed using Beckman Coulter ACT 5 diff hematology analyzer (Beckman Coulter Eurocenter, SA). Biochemistries were performed on cryopreserved plasma samples for glucose, lactate, lactate dehydrogenase (LDH), total (t-) bilirubin, creatinine, and blood urea nitrogen (BUN) using Roche Cobas Integra 400 plus Chemistry analyzer by the Advanced Research & Diagnostic Lab at the University of Minnesota. Acute kidney injury was defined as a 1.5-fold increase in creatinine level from estimated baseline, using the Kidney Disease: Improving Global Outcomes (KDIGO) criteria based on a

© 2021 Datta D et al. *JAMA Network Open.*

single admission creatinine measure.[6] Baseline creatinine was estimated using the population of healthy community children.

5. Immunoassays

Samples for analysis were marked only with a participant study ID and testing for all biomarkers was performed blind to all cohort affiliation. Ten percent of samples were run in duplicate to evaluate reproducibility of results. Plasma tau testing was performed from August to September, 2018, on an HD-1 analyzer at the Quanterix headquarters in Lexington, MA. Samples were thawed at room temperature and then mixed thoroughly until visibly homogenous following gentle inverting. Samples were spun for 30 seconds to remove any liquid from the caps. 100 µL were transferred to 1.7-mL microcentrifuge tubes pre-labeled with barcodes corresponding to the original sample tubes. Samples were then centrifuged at 20,000*g* for 3 minutes at 4C and then transferred to 96-well plates for testing. The HD-1 Analyzer automatically diluted the samples 4-fold in sample diluent. Testing for the endothelial activation markers (Von Willebrand factor [vWF], Angiopoietin [Angpt]-1 and Angpt-2, soluble P-selectin and E-selectin, soluble intercellular adhesion molecule-1 [sICAM-1] and vascular cellular adhesion molecule-1[sVCAM-1]) were done by ELISA (vWF, Angpt-1, Angpt-2) or cytometric bead assay using the Bio-Plex 200 system (soluble ICAM-1, VCAM-1, P- and E-selectin), as described previously.[7]

© 2021 Datta D et al. *JAMA Network Open.*

eTable 1. Admission CSF and Plasma Tau Concentrations in Children Younger Than 5 Years and 5 Years or Older

	Sample	N	<5 years of age	N	≥5 years of age	P value[a]
Cerebral malaria	CSF	116	483.11 (259.17, 1051.67)	29	217.72 (181.48, 406.99)	<.001
	Plasma	137	8.12 (4.39, 13.21)	45	5.90 (3.93, 7.63)	.03
Severe malarial anemia	Plasma	132	5.69 (3.26, 8.96)	30	4.87 (1.98, 7.27)	.30
Community controls	Plasma	102	2.47 (1.42, 3.81)	21	2.87 (2.22, 4.08)	.40

Data presented as median (interquartile range)
[a] Two-sample Wilcoxon rank-sum test.

© 2021 Datta D et al. *JAMA Network Open.*

eTable 2. Distribution of Parasite Factors, Clinical Risk Factors, and Levels of Clinical Lab Markers by Normal vs. Elevated Admission Tau Concentrations in Children with Cerebral Malaria and Severe Malarial Anemia

	Cerebral Malaria			Severe Malarial Anemia		
	Normal tau (n=82)[a]	Elevated tau (n=100)[a]	P value[b]	Normal tau (n=93)[a]	Elevated tau (n=69)[a]	P value[b]
**Parasite factors, Median (IQR)**						
Parasite density, parasites/uL	31250 (7800, 124400) (80)	53640 (16220, 294500) (98)	.03	24840 (8580, 91900)	47020 (14120, 178020)	.04
Plasma PfHRP-2, ng/mL	2192.4 (880.8, 1243.2)	3711.6 (1243.2, 6527.5)	.01	880.8 (283.2, 2577.6)	1010.4 (468.6, 2958.4)	.21
**Clinical risk factors, n (%)**						
Hyperpyrexia	6 (7.32)	4 (4)	.33	4 (4.3)	3 (4.4)	.66
Retinopathy, n (% positive)	51 (66.23)	62 (65.26)	.89	--	--	--
Multiple seizures	39 (47.56)	45 (45)	.73	1 (1.08)	0 (0)	.39
Deep breathing	2 (2.44)	12 (12)	.02	6 (6.5)	3 (4.4)	.56
Hypoxia	17 (20.73)	31 (31)	.12	17 (18.3)	14 (20.6)	.71
Lactic acidosis	24 (31.17)	33 (36.67)	.68	42 (49.4)	35 (53.9)	.59
Acute kidney injury	21 (26.6)	45 (47.9)	.004	12 (13.5)	17 (25.4)	.06
Uremia	23 (28.05)	49 (49)	.004	21 (22.6)	19 (27.5)	.47
Thrombocytopenia	65 (80.25)	91 (91.92)	.02	37 (39.8)	42 (61.8)	.006
**Clinical lab tests, Median (IQR)**						
Glucose, mmol/L	7.1 (5.6, 10)	6.65 (4.8, 9.05)	.23	6.8 (5, 8)	5.7 (4.7, 8.2)	.51
Hemoglobin, g/dL	6.3 (5.3, 8.6)	6.5 (5.3, 8.1)	.76	3.9 (3.2, 4.5)	3.9 (3.4, 4.5)	.83
LDH, U/L	746 (550.5, 901) (80)	905.5 (712, 1166.5) (92)	<.001	696 (556, 863) (89)	795.5 (684, 1140) (66)	.002
T-bilirubin, mg/dL	1.4 (0.8, 2) (81)	1.8 (0.9, 3.1) (92)	.07	1.1 (0.5, 1.8)	1.4 (0.8, 2.7)	.03

[a]N for missing data-points for any category listed in brackets
[b]Two-sample Wilcoxon rank-sum test for parasite factors and clinical lab tests, presented as median (IQR). Pearson's chi-square test for clinical risk factors presented as n (%) unless otherwise indicated.
Abbreviations: IQR, interquartile range; PfHRP-2, *Plasmodium falciparum* histidine-rich protein-2; LDH, lactate dehydrogenase; T-bilirubin, total bilirubin; --, not applicable

© 2021 Datta D et al. *JAMA Network Open.*

eFigure. Multivariate Logistic Regression Analysis of Factors Associated with Normal vs. Elevated Admission Tau Concentrations in Children with Cerebral Malaria and Severe Malarial Anemia. Model adjusted for age, sex and factors with a P<.05 in univariate logistic model in Figure 4A (PfHRP-2, acute kidney injury, thrombocytopenia, LDH, T-bilirubin).

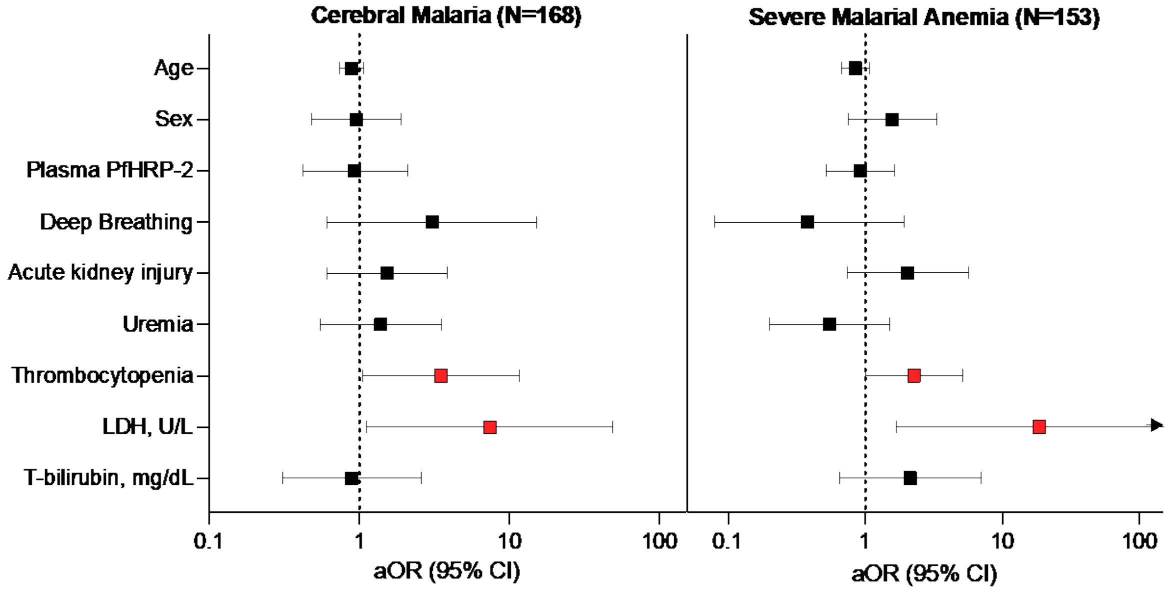

Abbreviations: aOR, adjusted odds ratio; PfHRP-2, *Plasmodium falciparum* histidine-rich protein-2; LDH, lactate dehydrogenase; T-bilirubin, total bilirubin.

© 2021 Datta D et al. *JAMA Network Open.*

References

1. Bradley RH, Caldwell BM, Corwyn RF. The Child Care HOME Inventories: assessing the quality of family child care homes. *Early Childhood Research Quarterly.* 2003;18(3):294-309.
2. Bangirana P, John CC, Idro R, et al. Socioeconomic predictors of cognition in Ugandan children: implications for community interventions. *PloS one.* 2009;4(11):e7898.
3. Opoka RO, Bangirana P, Idro R, Shabani E, Namazzi R, John CC. Lack of mortality in 22 children with sickle cell anemia and severe malarial anemia. *Pediatr Blood Cancer.* 2018;65(1).
4. Villaverde C, Namazzi R, Shabani E, Opoka RO, John CC. Clinical Comparison of Retinopathy-Positive and Retinopathy-Negative Cerebral Malaria. *Am J Trop Med Hyg.* 2017;96(5):1176-1184.
5. Park GS, Opoka RO, Shabani E, Wypyszynski A, Hanisch B, John CC. Plasmodium falciparum Histidine-Rich Protein-2 Plasma Concentrations Are Higher in Retinopathy-Negative Cerebral Malaria Than in Severe Malarial Anemia. *Open Forum Infect Dis.* 2017;4(3):ofx151.
6. KDIGO Clinical Practice Guideline for Acute Kidney Injury. Working Group. *Kidney Int.* 2012.
7. Ouma BJ, Ssenkusu JM, Shabani E, et al. Endothelial Activation, Acute Kidney Injury, and Cognitive Impairment in Pediatric Severe Malaria. *Crit Care Med.* 2020.

© 2021 Datta D et al. *JAMA Network Open.*
